# Targeting of Secretory Proteins as a Therapeutic Strategy for Treatment of Nonalcoholic Steatohepatitis (NASH)

**DOI:** 10.3390/ijms21072296

**Published:** 2020-03-26

**Authors:** Kyeongjin Kim, Kook Hwan Kim

**Affiliations:** 1 Department of Biomedical Sciences, College of Medicine, Inha University, Inha-ro 100, Michuhol-gu, Incheon 22212, Korea; 2 Metabolic Diseases Research Center, GI Cell, Inc., B-1014, Tera Tower, Songpa-daero 167, Songpa-gu, Seoul 05855, Korea

**Keywords:** NAFLD, NASH, secretory proteins

## Abstract

Nonalcoholic steatohepatitis (NASH) is defined as a progressive form of nonalcoholic fatty liver disease (NAFLD) and is a common chronic liver disease that causes significant worldwide morbidity and mortality, and has no approved pharmacotherapy. Nevertheless, growing understanding of the molecular mechanisms underlying the development and progression of NASH has suggested multiple potential therapeutic targets and strategies to treat this disease. Here, we review this progress, with emphasis on the functional role of secretory proteins in the development and progression of NASH, in addition to the change of expression of various secretory proteins in mouse NASH models and human NASH subjects. We also highlight secretory protein-based therapeutic approaches that influence obesity-associated insulin resistance, liver steatosis, inflammation, and fibrosis, as well as the gut–liver and adipose–liver axes in the treatment of NASH.

## 1. Introduction

Nonalcoholic fatty liver disease (NAFLD)/nonalcoholic steatohepatitis (NASH) are chronic liver diseases with significant worldwide health implications [1]. NAFLD compromises a wide range of histologic manifestations such as simple fatty liver (steatosis), NASH, and eventually advanced fibrosis (cirrhosis) with irreversible scarring features [1]. Human populations with simple steatosis (intrahepatic fat accumulation exceeding 5% of total liver weight) is approximately 15% to 40%. Among those people with simple steatosis, 10%–20% progress to NASH with features of hepatic inflammation and fibrosis [2]. Of patients with NASH, 15%–25% develop advanced fibrosis/cirrhosis [2]. Further, NASH predisposes individuals to obesity-associated insulin resistance, dyslipidemia, cardiovascular disease, and hepatocellular carcinoma (HCC) [2]. 

Growing evidence shows that secretory proteins are affected by various intracellular events involved in NASH, including endoplasmic reticulum (ER) stress, mitochondrial stress, lipotoxicity, reactive oxygen species (ROS) production, and intercellular events between different liver cell types [3,4,5]. Secretory changes can have either protective or detrimental systemic effects. Here, we briefly describe molecular mechanisms involved in pathogenesis of steatosis, inflammation, or fibrosis, highlighting contributions of each cell type (such as hepatocyte, Kupffer cell (KC), or hepatic stellate cell (HSC)) and crosstalk between these cell types within the liver. We also discuss the in vivo role of key secretory proteins in the development and progression of NASH. Finally, we illustrate therapeutic potential of secretory proteins in NASH.

## 2. Molecular Mechanisms Underlying Nonalcoholic Steatohepatitis (NASH) Pathogenesis 

The most recent understanding in NASH pathogenesis is that there are multiple hits, resulting in hepatocyte lipid accumulation, inflammation, and fibrosis [6]. In the following subsections, we briefly describe the putative molecular mechanisms underlying each of these NASH criteria, with focus on intracellular and intercellular events between hepatocytes, KCs/macrophages, and HSCs.

### 2.1. Steatosis/Cell Death (Hepatocytes)

Accumulation of lipid droplets in the cytoplasm of hepatocyte is an initiating step in development of NAFLD/NASH, attributable to an imbalance between lipid acquisition and lipid disposal. Steatosis occurs when (1) circulating free fatty acid (FFA) uptake is increased, (2) hepatic de novo lipogenesis (DNL) is elevated, (3) fatty acid β-oxidation (FAO) is decreased, and/or (4) very low-density lipoprotein (VLDL) lipid export is impaired [7]. Although the molecular mechanisms regulating hepatic lipid homeostasis in NALFD/NASH livers are not fully elucidated, each of these pathways likely impact hepatic steatosis. We review each below.

1. FFA uptake—Circulating FFAs released from adipose tissues by lipolysis can enter the liver, contributing to the largest amount of hepatic lipid. This process is mediated by cluster of differentiation 36 (CD36), caveolin, and fatty acid transport (FATP), all located in the plasma membrane of hepatocytes [8]. Although the role of hepatic caveolin is less well-studied, FATP isoforms (FATP2 and FATP5) and CD36 have been shown to participate in increased uptake of circulating FFAs and development of steatosis. Knockdown of FATP2, FATP5, or CD36 leads to decrease of hepatic FFA uptake and ameliorates hepatic steatosis in mice [9,10,11], supporting the fundamental importance of FATPs and CD36 to hepatic steatosis.
2. DNL—DNL is a process to synthesize new fatty acids from acetyl coenzyme A (acetyl-CoA). In the steatotic, insulin-resistant liver, although insulin-induced suppression of gluconeogenesis is impaired, insulin-stimulated DNL rates increase. This is referred to as selective insulin resistance [12]. Although molecular regulators of this paradox are not fully understood, recent results have suggested that PHLPP2 (pleckstrin homology domain leucine-rich repeat protein phosphatase 2), an Akt Ser473 phosphatase, acts as a novel regulator to terminate insulin-induced DNL, with no impact on early-postprandial gluconeogenesis [13,14]. Degradation of PHLPP2 in obese liver sustains Akt-mediated induction of sterol regulatory element-binding protein 1c (SREBP-1c) and DNL [7]. Consequently, increased DNL has been shown to contribute to the steatosis of NAFLD/NASH patients [15].
3. FAO—There have been conflicting reports on the role of FAO in NAFLD/NASH patients [16,17]. Although reduced FAO has been reported to contribute to increased hepatic lipid accumulation [17], increased markers of FAO have also been observed in steatotic livers, a likely compensatory mechanism to alleviate excessive lipid accumulation [16].
4. VLDL secretion—Export of lipids from the liver is an important pathway to reduce hepatic lipid accumulation. apoB100 and microsomal triglyceride transfer protein (MTP), key components in hepatic VLDL secretion, are negatively regulated by insulin [18]; however, selective insulin resistance in NAFLD patients allows insulin to stimulate DNL without suppressing VLDL production [19].

**Figure 1:**
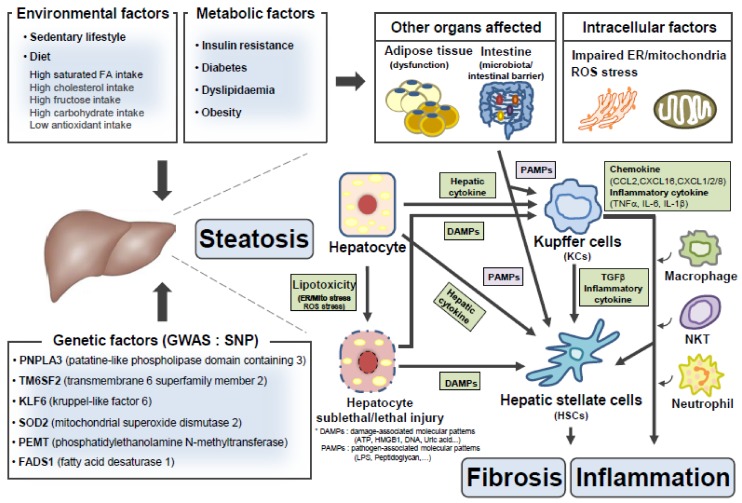
The “multiple-parallel hit” model in the pathogenesis of nonalcoholic steatohepatitis (NASH). Environmental, metabolic, and genetic factors participate in the development of steatosis and the progression to inflammation or fibrosis by affecting diverse cell types (hepatocyte, Kupffer cell (KC), or hepatic stellate cell (HSC)) in the liver and other tissues (the intestine or adipose tissue). Excess caloric intake (environmental factor) leads to obesity and insulin resistance (metabolic factor) to cause hepatic steatosis. Increased hepatic lipids and/or lipid metabolites cause oxidative or organelle stress, leading to hepatocyte sublethal/lethal injuries. Downstream factors (cytokines/chemokines or damage-associated molecular patterns (DAMPs)) derived from injured hepatocytes stimulate an inflammatory response in KCs and a fibrotic response in HSCs, leading to liver inflammation and fibrosis. Insulin resistance/diabetes and obesity (metabolic factor) also influence organ crosstalk between the liver and other tissues (the intestine/adipose tissue), contributing to the development and progression of NASH.

Overall, these pathways lead to intracellular lipid accumulation in hepatocytes. The proximal cause for many of these abnormalities is obesity-induced insulin resistance, in response to environmental factors (sedentary lifestyle or nutrient overload such as high-fat, high-carbohydrate, or high-fructose diets) that exacerbate underlying genetic predisposition (such as single nucleotide polymorphisms of *patatin-like phospholipase domain containing protein 3* (*Pnpla3*) or *transmembrane 6 superfamily member 2* (*Tm6sf2*), leading to hepatic lipid accumulation (Figure 1). In steatoic livers, increased excessive lipids or its metabolites subsequently cause excessive production of reactive oxygen species (ROS) and dysfunction of intracellular organelles (ER/mitochondria). These events culminate in hepatic sublethal or lethal injury (cell death) (Figure 1).

Therefore, pharmacologic agents that inhibit DNL and lipid-induced oxidative stress, or enhance FAO, are considered potential therapeutic candidates for treatment of NAFLD/NASH. For example, inhibitors of the lipogenic or triglyceride (TG) synthetic machinery such as fatty acid synthase (FAS) (TVB-2640; clinial trials NCT03938246 and FT-4101; NCT04004325), acetyl-CoA carboxylase (ACC) (firsocostat/GS-0976; NCT02856555 and PF-05221304; NCT03448172/NCT03248882), steroyl-CoA desaturase 1 (SCD) (aramchol; NCT04104321), and diacylglycerl O-actyltransferase 2 (DGAT2) (IONIS-DGAT2_rx_; NCT03334214 and PF-06865571; NCT03513588/NCT03776175) are in phase 1b, 2, or 3 clinical trials to reduce hepatic TG content in NASH patients. As therapeutic molecules to diminish oxidative stress, antioxidants (resveratrol or vitamin E) have been evaluated [20,21]. Nuclear hormone receptors that exert pleiotropic effects on hepatic lipid metabolism are also attractive targets for treatment of NASH [22]. Dual peroxisome proliferator-activated receptor α/γ (PPARα/γ) (saroglitazar, NCT03061721) and PPARα/δ agonist (elafibranor, NCT01694849, and NCT02704403) are in advanced phase 2/3 trials. Similarly, thyroid hormone receptor β (THRβ) agonists (VK2809; NCT04173065 and resmetirom/MGL-3196; NCT03900429) are also in phase 2/3 studies. Pharmacologic agents for blocking hepatic injury have fared less well in recent trials, including caspase (Emricasan/IDN-6556; NCT03205345) and apoptosis signaling kinase 1 (ASK-1) inhibitors (Selonsertib/GS-4997; NCT03053063), which did not meet NASH/cirrhosis primary endpoints. 

### 2.2. Inflammation (KCs/Macrophages and Other Immune Cells)

In the setting of NASH, lipotoxicity-induced hepatic sublethal or lethal injury is able to stimulate KCs to release hepatocyte-derived factors such as damage-associated molecular patterns (DAMPs), extracellular vesicles (exosomes), or inflammatory cyotokines [23]. Activated KCs secrete more pro-inflammatory cytokines and chemokines, leading to recruitment of other immune cells (such as macrophages, neutrophils, or natural killer T cells (NKTs)) and activation of HSCs [24] (Figure 1). Toll-like receptors (TLRs) are pattern recognition receptors (PRRs) that act as sensors of the innate immune system together with nucleotide-binding and oligomerization domain (NOD)-like receptors (NLRs). Endotoxin (lipopolysaccharide) and lipid metabolite (ceramide or palmitic acid) activates TLR or NLR signaling in hepatocytes and KCs/macrophages, leading to increased secretion of the inflammasome-activating cytokine IL-1β and pro-inflammatory cytokines/chemokines (TNFα, IL-6, CCL2, CXCL16 or CXCL1/2/8) [24,25]. Growing evidence has suggested that the inflammasome is a critical triggering factor for progression from steatosis to NASH [26,27]. Preclinical animal studies using a small molecule NLR family pyrin domain containing 3 (NLRP3) inhibitor (MCC950) or mice with deletion of *Il-1β* or *Il-1r* have revealed that deficiency of the inflammasome results in reduced hepatic inflammation and fibrosis in mice [26,27]. These data suggest that inflammasome could be a potential target for treatment of NAFLD/NASH. Similarly, the chemokine C-C chemokine receptor 2/5 (CCR2/CCR5) inhibitor (cenicriviroc) suppresses recruitment of monocytes and activation of HSCs, and is currently being evaluated in a phase 3 clinical study (NCT03028740) in NASH patients with liver fibrosis. An amine oxidase copper-containing 3 (AOC3) inhibitor (BI1467335/PXS-4728A) is also in a phase 2 clinical trial (NCT03166735) to block infiltration of immune cells in the liver.

Inflammation in other tissues, such as adipose tissue and intestine, may contribute to the development and progression of NAFLD/NASH [28,29]. Inflammation in white adipose tissue (WAT) can induce hepatic inflammation [28]. Neutrophils or macrophages infiltrating in WATs produce pro-inflammatory mediators or cytokines, which contribute to systemic inflammation [30,31]. Further, loss of protective adipokines (such as adiponectin and leptin) secreted from WATs may affect lipid accumulation, inflammation, and fibrosis in the liver [32] (Figure 1). In addition to WAT, decreased brown adipose tissue (BAT) activity has been associated with the development and progression of NAFLD [33,34]. Combined therapy of BAT activation (treatment of β3AR agonist) and caloric restriction synergistically improve NASH in an animal model, although BAT activation alone does not reverse NASH despite alleviation of steatosis [34]. 

The intestine–liver axis similarly participates in pathogenesis of NASH [29,35]. It has been reported that microbiota composition is changed and intestine barriers are disrupted during the progression of NAFLD/NASH [36,37,38]. Consequently, microbiota-derived endotoxin may enter the liver to activate hepatic inflammation via stimulation of TLRs and NLRs (Figure 1). The importance of the gut–liver axis in pathogenesis of NAFLD/NASH has been recently reviewed [29,35]. From a pharmacologic standpoint, the farnesoid X receptor (FXR) is expressed in the ileum as well as in hepatic parenchymal and nonparenchymal cells [39]. FXR activation exerts pleiotropic effects in intestinal enterocytes, hepatocytes, KCs, or HSCs [39,40], which results in improvement in steatosis, inflammation, and fibrosis in preclinical NASH animal models [39,40]. Thus, several FXR agonists, such as obeticholic acid (NCT01265498, NCT02548351 and NCT03439254), GS-9674 (NCT02854605 and NCT03449446), tropifexor (NCT02855164), and EDP-305 (NCT02918929 and NCT03421431) are in trials for NASH therapeutics.

### 2.3. Fibrosis (HSCs) 

Liver fibrosis features the accumulation of large amounts of extracellular matrix (ECM) proteins such as collagen or fibronectin. Liver myofibroblasts, originating from HSCs, portal fibroblast (PFs), and mesothelial cells, play a crucial role in the progression of fibrosis [41]. Activation of HSCs largely involves the transition from quiescent phenotypes to proliferative, migratory, and fibrogenic (myofibroblast-like) features, characteristic of NASH-related fibrosis. HSCs are activated via the crosstalk between multiple cell-surface, cytoplasmic, and nuclear signal molecules/pathways [41], including high mobility group box 1 (HMGB1), Hedgehog, Notch, and Yes-associated protein/transcriptional coactivator with PDZ-binding motif (YAP/TAZ) pathways [42,43,44,45,46]. In addition, HSCs are activated by extracellular/paracrine signals from surrounding cells such as hepatocytes, macrophages, natural killer cells, natural killer T cells, B cells, liver sinusoidal endothelial cells (LSECs), and platelets [47].

Several HSC-targeted therapies have been tested in NASH patients. Lysyl oxidase-like 2 (LOXL2) is an enzyme that catalyzes collagen cross-linking to remodel the extracellular matrix, leading to development of a monoclonal antibody against LOXL2 (Simtuzumab/GS-6624). However, efficacy of simtuzumab as a monotherapy was minimal [48]. Galectin-3, a lectin derived from KCs/macrophages, plays an important role in transforming growth factor beta (TGFβ)-mediated activation of HSCs [49], leading to phase 3 trial for a galectin-3 inhibitor (GR-MD-02).

## 3. Secretory Proteins as Therapeutic Targets for NASH

### 3.1. Incretins

Glucagon-like protein 1 (GLP-1) is an incretin hormone derived from proglucagon produced in the intestine, together with glucose-dependent insulinotropic polypeptide (GIP). GLP-1 plays an important role in adaption to nutrient changes [50]. In response to feeding, intestinal GLP-1 production enhances pancreatic insulin release to decrease blood glucose, but also inhibits gastric emptying to suppress food intake [50]. GLP-1 can additionally increase lipid catabolism via enhancement of β-oxidation or thermogenesis, and also inhibit lipid accumulation via suppression of de novo lipogenesis, leading to improvements of diet-induced obesity and insulin resistance in mice [51,52]. Synthetic GLP-1 receptor agonists (exenatide, liraglutide, dulaglutide, or semaglutide) are available for treatment of type 2 diabetes and obesity. Intriguingly, GLP-1 agonists also attenuate hepatic inflammation and fibrosis as well as hepatic steatosis in mice [53,54]. Liraglutide has also been shown to reduce DNL, as well as improve hepatic steatosis in NASH patients [55], suggesting that GLP-1 agonists could be repurposed as NASH therapeutics. Inhibitors of dipeptidylpeptidase 4 (DPP4), an enzyme degrading GLP-1 (sitagliptin or vildagliptin) have also been shown to reduce serum liver enzyme levels and hepatic lipid accumulation in a NASH mouse model [56] or human NAFLD (or NASH) patients [57]. In addition, GLP-1/glucagon (GCG) receptor dual agonists or GLP-1/GCG/GIP receptor triple agonists are attractive NAFLD/NASH therapeutics, due to improved effects on diet-induced metabolic deterioration and steatohepatitis in mice [58,59,60,61]. On the basis of the importance of incretin-based preclinical therapeutics, phase 1/2 clinical studies using GLP1 analogs (liraglutide or semaglutide), GLP-1/GCG receptor dual agonists (cotadutide/MEDI0382), GLP-1/GIP receptor dual agonists (trizepatide/LY3298176 or CT868), and GLP-1/GCG/GIP receptor triple agonists (HM15211) are being conducted in human NASH patients (Table 1).

### 3.2. Growth Differentiation Factor (GDF15) Agonists 

GDF15 belongs to the transforming growth factor β (TGFβ) superfamily, and has been identified as a secretory protein with predominant expression in liver, placenta, and macrophages [62]. However, GDF15 expression has also been reported in adipose tissue, muscle, lung, kidney, and heart [62]. Growing evidence has suggested that GDF15 is induced in the livers of mice or human subjects with various liver injuries including NAFLD/NASH, hepatic viral/bacterial infections, and HCC [63,64,65]. For example, GDF15 expression is increased in the livers of mice fed a NASH-provoking diet (methionine-choline-deficient (MCD) or amylin liver NASH model (AMLN)) and in human subjects with NASH or advanced fibrosis [66]. GDF15 expression is also increased in the livers of mice with alcohol feeding or human subjects with alcoholic steatohepatitis (ASH) [67], suggesting that GDF15 may be a biomarker for common liver diseases. Interestingly, treatment with recombinant GDF15 or genetic overexpression of GDF15 resulted in improvement of inflammatory and fibrotic features in mice fed an ASH or NASH diet [66,67]. Consistent with this data, GDF15 has been recently reported to exert an anti-fibrotic action in other tissues such as kidney and lung [68,69]. Despite these preclinical studies, clinical trials of GDF15 for NASH have not been performed (Table 1).

### 3.3. Fibroblast Growth Factor 15 (FGF15)/FGF19 Agonists

FGF15/FGF19 (FGF15 in mice and FGF19 in humans) is a hormone produced in the intestine that plays a crucial role in the regulation of bile acid metabolism in the liver [70]. Feeding rapidly induces the release of bile acids stored in the gallbladder to help digestion and absorption of fats. In the late-postprandial state, small intestine enterocyte-derived FGF15/FGF19 enters the liver via the portal vein, where it binds its receptor (FGFR4) in hepatocytes to repress de novo bile acid synthesis through suppression of cholesterol 7a-hydroxylase (CYP7A1), a rate-limiting enzyme for conversion of cholesterols to bile acids [70]. Intriguingly, FGF15/FGF19 also has potent effects on lipid or glucose metabolism [71,72]. FGF15/FGF19 stimulates glycogen synthesis and suppresses gluconeogenesis, similar to the action of insulin in the liver [71]; however, in contrast to insulin, FGF15/FGF19 decreases hepatic TG accumulation [72]. In addition, FGF15/FGF19 may enhance insulin sensitivity by multiple actions, including increased β-oxidation, reduced lipogenesis, or diminished lipotoxicity [73,74]. Thus, FGF15/FGF19 is a promising therapeutic molecule for treatment of obesity-related metabolic deterioration. Intriguingly, serum FGF19 level is reduced in patients with NAFLD/NASH, and its action in the liver is also impaired in these subjects [75,76]. Early phase 2 studies (NCT02443116) of aldafermin (NGM282, a nontumorigenic FGF19 variant) showed efficacy in reducing hepatic steatosis and hepatic inflammation/fibrosis in patients with NASH [77]. Further clinical studies (NCT03912532; NCT04210245) are currently underway to evaluate the efficacy of FGF15/FGF19 as a NASH therapeutic (Table 1).

### 3.4. FGF21 Agonists

FGF21 was identified as a secretory protein that enhances insulin action in adipocytes [78]. Growing evidence has suggested that FGF21 acts as a hormone that is able to regulate glucose or lipid metabolism in response to environmental stimuli or nutrient stresses [79]. FGF21 exerts beneficial effects on obesity and related metabolic diseases via multiple actions, such as enhancements of insulin-mediated glucose uptake and β-oxidation/thermogenesis, as well as amelioration of ER stress [78,80,81]. There results suggest the potential of FGF21 as a drug candidate to treat obesity-related type 2 diabetes. In a recent clinical study (NCT02097277) of pegbelfermin (BMS-986036, polyethylene glycol-attached, PEGylated FGF21), however, little effect on Hb1Ac or body weight was observed in obese patients with type 2 diabetes [82]. Nonetheless, FGF21 remains an attractive therapeutic target for treatment of NASH, as numerous preclinical studies have suggested that FGF21 alleviates steatohepatitis and fibrosis in NASH diet-fed mice via decrease of hepatic lipotoxicity, increase of β-oxidation, inhibition of fibrogenesis, or activation of an adiponectin-IL17A axis [83,84,85]. In several clinical studies (NCT02097277 or NCT02413372) for human NASH subjects, 12 weeks or 16 weeks of pegbelfermin led to alleviation of steatosis and improvements of metabolic parameters (HDL, TG, adiponectin) and fibrosis biomarkers [82,86]. Ph2b clinical studies (NCT03486899 and NCT03486912) of pegbelfermin in NASH patients with advanced fibrosis are currently underway (Table 1). In addition, phase 1/2 clinical trials of other FGF21 agonists (NCT03060538; agonistic anti-FGFR1/KLB antibody (BFKB8488A), NCT03298464; anti-FGFR1c/KLB antibody (MK-3655/NGM313), NCT03976401; an engineered Fc-FGF21 (AKR-001) and NCT04048135; a glycopegylated FGF21 (BIO89-100)) are underway or completed in patients with NAFLD or NASH (Table 1).

### 3.5. Mitochondrial Open Reading Frame of the 12S rRNA Type-c (MOTS-c) Agonists

MOTS-c, a naturally occurring mitochondrial peptide, has been identified as a regulator of metabolic homeostasis. Treatment with MOTS-c alleviates high-fat diet (HFD)-induced steatosis, obesity, and insulin resistance in mice [87]. The metabolic beneficial effect of MOTS-c is likely due to increased lipid catabolism and enhanced energy expenditure [87], but MOTS-c is also able to inhibit lipolysis in adipocyte in an insulin-independent manner, which contributes to reduced release of fatty acids from adipocytes and improvement of hepatic steatosis. Interestingly, treatment with MOTS-c resulted in improvements of serum alanine aminotransferase (ALT) level, hepatic TG content, and NAFLD activity score (NAS) in the stelic animal model (STAM) animal model, suggesting therapeutic potential of MOTS-c for treatment of NASH. A phase 1 clinical study (NCT03998514) of MOTS-c analogs (CB4211) in patients with NAFLD/NASH is currently underway (Table 1). Considering the role in energy metabolism of mitochondrial-derived peptides (MDPs) such as Humanin or small Humanin-like peptides 2 (SHLP2) [88,89], mitochondria-based therapeutics might be potential strategies for treatment of various human metabolic diseases including NAFLD/NASH. Further studies are needed to identify more novel MDPs and evaluate their functional role in pathogenesis of NAFLD/NASH and the possibility of their therapeutic application.

## 4. Conclusions

Despite the significant clinical impact of NAFLD/NASH, no therapeutic drugs are approved for its treatment. Most therapeutic strategies have focused on monotherapy targeting one among four main pathogenic processes (steatosis, hepatic sublethal/lethal injury, or inflammation or fibrosis). As discussed above, intracellular events (hepatic lipid accumulation or ER/mitochondria/ROS stress), inter-cell liver communication (between parenchymal and non-parenchymal cells), and inter-organ communications (adipose-liver axis or gut-liver axis) all participate in the development and progression of NAFLD/NASH. Given the value of a “multiple-hit parallel model” in the pathogenesis of NASH, strategies targeting two or more pathogenic processes in NASH may be more appropriate. Considering the action of secretory proteins in different liver cell types and in distant organs, therapeutic strategy with secretory proteins may bring about novel NASH therapeutic opportunities. In addition, combined therapy with agents targeting single pathogenic processes (e.g., FXR agonist (tropifexor) and CCR2/5 antagonist (cenicriviroc)) could be coupled with novel entries (e.g., GLP1-IgG Fc-FGF21) to produce an efficacious therapeutic approach for treatment of NASH. Finally, combined therapeutic approaches based on chemicals, biologics, antisense, or microbiome might also be attractive. Further preclinical and clinical testing allow for more valuable approaches for treatment of NAFLD/NASH.

## Figures and Tables

**Table 1:** Secretory protein-based pharmacological agents under development for treatment of nonalcoholic fatty liver disease (NAFLD)/NASH.

Drugs	Target of Action	Company	Highest Developmental Stage
Liraglutide	GLP-1 receptor agonist	Novo Nordisk	Phase 2
Semaglutide	GLP-1 receptor agonist	Novo Nordisk	Phase 2
Cotadutide/MEDI0382	GLP-1/GCG receptor dual agonist	AstraZeneca/MEDIMMUNE	Phase 2
CT-868	GLP-1/GIP receptor dual agonist	Carmot Therapeutics	Phase 1
Trizepatide/LY3298176	GLP-1/GIP receptor dual agonist	Eli Lilly	Phase 2
HM15211	GLP-1/GCG/GIP receptor triple agonist	Hanmi Pharmaceutical	Phase 1
Aldafermin (NGM282)	FGF19 analog	NGM Biopharmaceuticals	Phase 2b
Pegbelfermin (BMS-986036)	FGF21 analog (PEGylated FGF21)	Bristol-Myers Squibb/ Ambrx	Phase 2b
AKR-001	Fc-FGF21	Akero	Phase 2
BIO89-100	FGF21 analog (glycoPEGylated FGF21)	89bio	Phase 1b/2a
BFKB8488A	Agonistic anti-FGFR1/KLB antibody	Genentech, Inc.	Phase 1
NGM313/ MK-3655	Agonistic anti-FGFR1c/KLB antibody	Merck/ NGM Biopharmaceuticals	Phase 1
YH25724	GLP-1/FGF21 dual agonist	Boehringer Ingelheim GmbH/Yuhan Corporation	Pre-clinical phase
CB4211	MOTS-c analogs	CohBar, Inc.	Phase 1
NGM395 *	GDF15 analogs	NGM Biopharmaceuticals	N/A (not applicable)
GDF15 Agonist **	GDF15 analogs	Eli Lilly	N/A
LA GDF15 ***	GDF15 analogs	Novo nordisk	N/A

* Phase 1 for obesity/NAFLD; ** phase 1 for type 2 diabetes; *** phase 1 for obesity.
